# Biopolymer-Based Active and Intelligent Food Packaging: Recent Advances in Materials, Technologies, and Applications

**DOI:** 10.3390/polym18020196

**Published:** 2026-01-10

**Authors:** Shakila Parveen Asrafali, Thirukumaran Periyasamy, Jaewoong Lee

**Affiliations:** Department of Fiber System Engineering, Yeungnam University, Gyeongsan 38541, Republic of Korea; shakilaparveen@yu.ac.kr (S.P.A.); thirukumaran@ynu.ac.kr (T.P.)

**Keywords:** biopolymer packaging, active packaging, intelligent packaging, food preservation, antimicrobial films, pH indicators, polysaccharides, nanocomposites, sustainable packaging

## Abstract

The food packaging industry is undergoing a paradigm shift from conventional petroleum-based materials toward sustainable biopolymer-based alternatives that offer enhanced functionality beyond mere containment and protection. This comprehensive review examines recent advances in the development of active and intelligent food packaging systems utilizing natural biopolymers including polysaccharides, proteins, and their composites. The integration of antimicrobial agents, natural colorimetric indicators, nanofillers, and advanced fabrication techniques has enabled the creation of multifunctional packaging materials capable of extending shelf life, monitoring food quality in real-time, and reducing environmental impact. This review organizes the current research on starch, chitosan-, cellulose-, pectin-, bacterial cellulose-, pullulan-, gelatin-, zein-, and dextran-based packaging systems, with particular emphasis on their physicochemical properties, functional performance, and practical applications for preserving various food products, including meat, fish, fruits, and other perishables. The challenges associated with mechanical strength, water resistance, scalability, and commercial viability are critically evaluated alongside emerging solutions involving chemical modifications, nanocomposite formulations, and innovative processing technologies. Future perspectives highlight the need for standardization, life cycle assessments, regulatory frameworks, and consumer acceptance studies to facilitate the transition from laboratory innovations to industrial-scale implementation of sustainable biopolymer packaging solutions.

## 1. Introduction

The global food packaging industry faces unprecedented challenges in the twenty-first century, driven by escalating environmental concerns regarding plastic waste accumulation, increasing consumer demand for safe and high-quality food products, and stringent regulatory requirements for sustainable materials. Traditional petroleum-derived packaging materials, while offering excellent barrier properties and mechanical strength, contribute significantly to environmental pollution due to their non-biodegradable nature and persistence in ecosystems for hundreds of years. Simultaneously, approximately one-third of all food produced globally is lost or wasted annually, representing not only a significant economic burden but also a substantial waste of the natural resources, including water, land, and energy, invested in food production. These converging pressures have catalyzed intensive research efforts toward developing sustainable packaging alternatives that can effectively preserve food quality while minimizing environmental footprint [1,2,3,4].

Biopolymer-based packaging materials have emerged as promising candidates to address these multifaceted challenges, offering dual advantages of biodegradability and functionality derived from renewable resources. Natural biopolymers, including polysaccharides such as starch, chitosan, cellulose, pectin, pullulan, and dextran, as well as proteins like gelatin and zein, possess inherent properties that make them attractive for food packaging applications. These materials demonstrate excellent film-forming capabilities, biocompatibility, and the ability to serve as matrices for incorporating active compounds such as antimicrobial agents, antioxidants, and colorimetric indicators. The concept of active packaging extends beyond the passive barrier function of conventional materials by incorporating components that interact with food or headspace environments to extend shelf life, maintain quality, and enhance safety [5,6,7]. Intelligent or smart packaging takes this functionality further by incorporating sensing elements that provide real-time information about food quality, freshness, or storage conditions through visible color changes or other detectable signals.

Recent advances in materials science, nanotechnology, and processing techniques have significantly expanded the possibilities for designing sophisticated biopolymer packaging systems with tailored properties. The incorporation of nanofillers such as cellulose nanocrystals, chitin nanofibers, metal–organic frameworks, and nanoparticles have proven effective in overcoming the inherent limitations of biopolymers, particularly their poor mechanical strength and high water vapor permeability. Chemical modifications including esterification, cross-linking, and grafting have enabled fine-tuning of hydrophobicity, crystallinity, and functional group availability. Advanced fabrication technologies, such as electrospinning, solution blow spinning, and three-dimensional cryogel formation, have opened new avenues for creating nanostructured materials with enhanced surface area and controlled release properties. The synergistic combination of multiple biopolymers in composite and multilayer structures has demonstrated superior performance compared to single-component systems, leveraging the complementary properties of different materials [8,9,10].

This review provides a comprehensive analysis of recent developments in biopolymer-based active and intelligent food packaging, with particular focus on the polysaccharide- and protein-based systems developed over the past few years. The discussion encompasses the fundamental properties of various biopolymers, strategies for enhancing their functionality through the incorporation of active agents and nanofillers, advanced processing and modification techniques, performance evaluation in preserving different food products, and a critical assessment of challenges and future directions. By synthesizing findings from diverse research efforts, this review aims to provide insights into the current state of the field and identify promising pathways toward commercial realization of sustainable, functional biopolymer packaging solutions.

## 2. Polysaccharide-Based Packaging Systems

Polysaccharides represent the most extensively studied class of biopolymers for food packaging applications, owing to their abundance, renewability, film-forming properties, and diverse structural characteristics that can be tailored for specific applications. The following sections examine recent advances in packaging systems based on major polysaccharide types, including their modifications, composite formulations, and functional enhancements [7,8,9].

### 2.1. Starch-Based Packaging Materials

Starch, a naturally abundant and cost-effective polysaccharide derived from various botanical sources, including corn, potato, cassava, and rice, has attracted considerable attention as a base material for biodegradable packaging films. Native starch consists of two glucose polymers, amylose and amylopectin, whose ratio and molecular characteristics significantly influence the properties of the resulting films. Recent research has focused on overcoming the inherent limitations of starch-based materials, particularly their poor mechanical properties, high water sensitivity, and limited antimicrobial activity, through various modification strategies and composite formulations.

**Figure 1:**
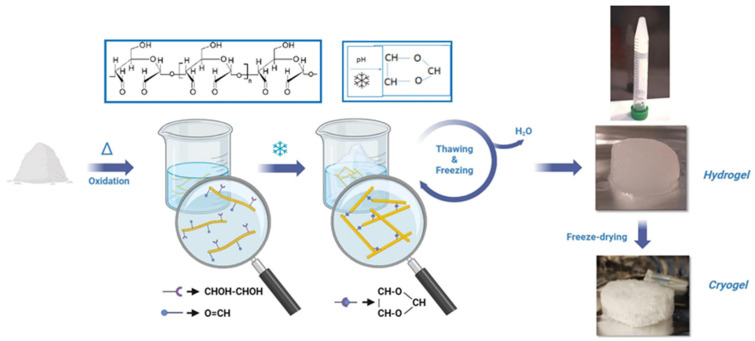
Schematic overview and experimental design for obtaining hydrogels and cryogels [15].

The development of antimicrobial starch-based cryogels and hydrogels represents a significant advancement in creating dual-active packaging systems that combine structural functionality with bioactive properties (Figure 1). Cryogelation, a process involving polymerization or cross-linking at sub-zero temperatures, produces materials with unique macroporous structures that exhibit excellent mechanical properties, shape recovery capabilities, and controlled release characteristics. In recent investigations, starch-based cryogels have been engineered to incorporate antimicrobial agents, such as essential oils, natural extracts, or antimicrobial peptides, within their three-dimensional network structure. The macroporous architecture of cryogels facilitates controlled diffusion of antimicrobial compounds to the food surface while maintaining structural integrity under various storage conditions. These materials demonstrate significant potential for extending the shelf life of perishable foods by inhibiting microbial growth through sustained release of bioactive compounds. The dual-active nature of these systems refers to their ability to provide both physical protection through their structural properties and biochemical protection through antimicrobial activity. The cryogel format offers advantages over conventional films in terms of cushioning properties, making them particularly suitable for packaging delicate food products that require both antimicrobial protection and mechanical support [11,12,13,14,15].

Hydrogel formulations of starch, created through chemical or physical cross-linking in aqueous environments, provide alternative architectures with high water content and tunable swelling properties. These materials can be designed to respond to environmental stimuli such as pH, temperature, or humidity, enabling smart packaging applications where the material properties change in response to food spoilage indicators. The incorporation of antimicrobial agents into starch hydrogels has been achieved through various strategies, including direct mixing, layer-by-layer assembly, and encapsulation in nanocarriers [16,17,18]. The hydrophilic nature of starch hydrogels facilitates intimate contact with high-moisture food products, enhancing the transfer of antimicrobial compounds to the food surface where microbial contamination typically initiates.

The development of multifunctional starch–pectin composite films represents another innovative approach to enhancing the performance of starch-based packaging. Pectin, a structural heteropolysaccharide found in plant cell walls, possesses excellent film-forming properties, biodegradability, and natural antimicrobial activity. The combination of starch and pectin in composite films leverages the complementary properties of both polysaccharides, with pectin contributing improved mechanical strength, water resistance, and bioactivity, while starch provides cost-effectiveness and good processability. Recent research has demonstrated that the incorporation of advanced functional fillers into starch–pectin matrices can create packaging materials with multiple capabilities including real-time monitoring and active preservation. One particularly innovative development involves engineering starch–pectin films with zeolitic imidazolate framework-67 (ZIF-67) loaded onto microcrystalline cellulose carriers [19,20,21,22]. This sophisticated nanocomposite approach addresses multiple packaging requirements simultaneously. ZIF-67, a cobalt-based metal–organic framework (MOF) composed of cobalt ions coordinated with 2-methylimidazole ligands, exhibits a distinct color-changing mechanism that makes it suitable for use as a colorimetric freshness indicator in intelligent food packaging. Its vibrant purple color originates from the d–d electronic transitions of the Co^2+^ ions within the framework. During food spoilage, nitrogen-containing volatile compounds such as ammonia, trimethylamine, and other amines are released, increasing the environmental alkalinity and interacting with the cobalt centers. These interactions cause ligand exchange or coordination changes between Co^2+^ and the amine molecules, altering the electronic structure of the metal ions and leading to a visible color shift—typically from purple to blue or pink depending on the coordination environment. In addition to serving as a colorimetric signal, ZIF-67 can release antimicrobial Co^2+^ ions, inhibiting bacterial growth and extending food shelf life. Thus, it functions dually as both a sensor and a preservative component in smart packaging systems for real-time spoilage detection. Microcrystalline cellulose serves as a carrier platform that improves the dispersion of ZIF-67 throughout the polymer matrix and contributes to enhanced mechanical properties through its rigid crystalline structure and ability to form hydrogen bonds with the polysaccharide chains.

**Figure 2:**
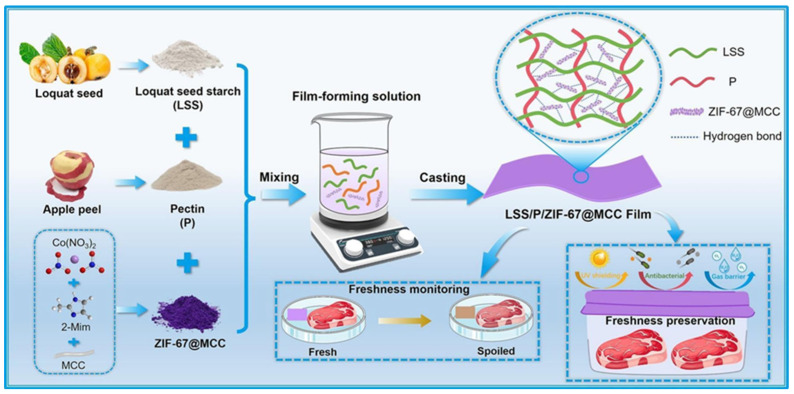
Schematic representation showing the synthesis of multifunctional starch/pectin composite film real-time monitoring and preserving of pork freshness [26].

**Figure 3:**
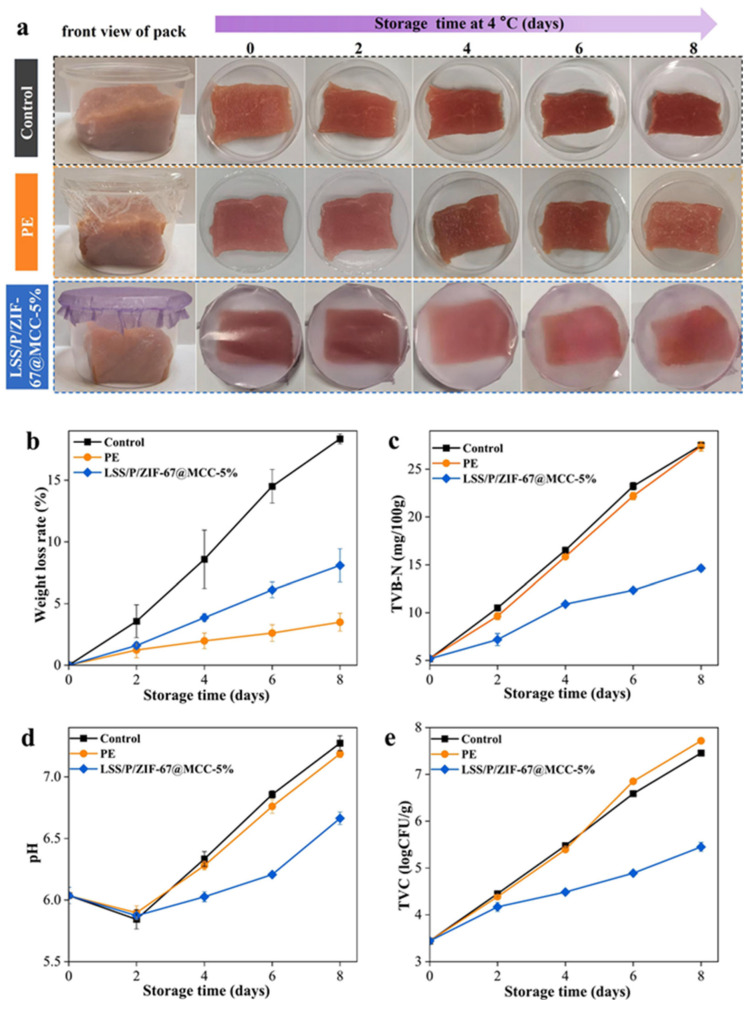
The changes in (**a**) visual appearance, (**b**) weight loss rate, (**c**) TVB-N, (**d**) pH, and (**e**) TVC values of pork wrapped with different packaging materials during storage at 4 °C [26].

The application of such multifunctional starch–pectin–ZIF-67 microcrystalline cellulose composites for preserving pork freshness demonstrates the practical viability of intelligent active packaging systems, represented in Figure 2 and Figure 3. Pork, being a highly perishable protein-rich food, undergoes rapid quality degradation through microbial growth, lipid oxidation, and protein decomposition, producing volatile nitrogen-containing compounds that serve as spoilage indicators. The composite film provides antimicrobial protection through ZIF-67, mechanical support through the starch–pectin–cellulose matrix, and real-time quality monitoring through visible color changes correlated with spoilage compound accumulation. Such integrated functionality represents a significant advancement over conventional packaging that requires separate monitoring systems or destructive testing to assess food quality. The development of these sophisticated starch-based composites illustrates the potential for creating economically viable packaging solutions that combine sustainability with advanced functionality, addressing both environmental concerns and food safety requirements [23,24,25,26].

### 2.2. Chitosan-Based Packaging Systems

**Figure 4:**
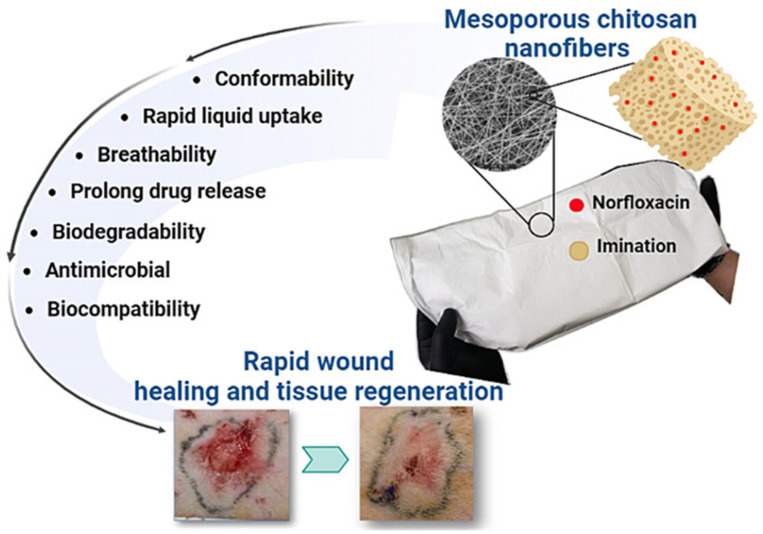
Mesoporous chitosan nanofibers loaded on bioabsorbable active dressings to accelerate the healing of burn wounds [38].

**Figure 5:**
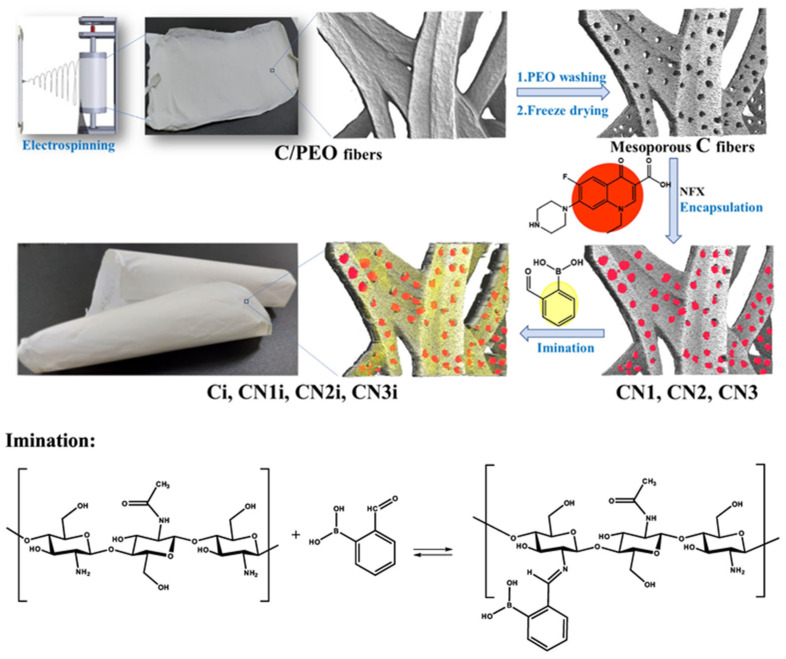
Preparation pathway of mesoporous chitosan composite fiber mats [38].

Chitosan, a cationic polysaccharide derived from chitin through deacetylation, stands out among biopolymers due to its inherent antimicrobial activity, excellent film-forming capability, and unique chemical structure amenable to various modifications. It exhibits potent antibacterial activity through multiple interrelated mechanisms that disrupt bacterial cell integrity and function. Its cationic nature—arising from positively charged amino groups in acidic environments—enables strong electrostatic interactions with negatively charged components of bacterial cell membranes, such as lipopolysaccharides and phospholipids. This interaction leads to membrane disruption, increased permeability, and leakage of intracellular contents, ultimately causing cell death. Additionally, chitosan can bind to bacterial DNA, inhibiting RNA synthesis and protein production, which interferes with essential metabolic processes. In some cases, low-molecular-weight chitosan penetrates cell walls and interacts directly with intracellular targets, while high-molecular-weight chitosan forms a polymer film on the bacterial surface, blocking nutrient transport. Chitosan also acts as a metal ion chelator, depriving bacteria of essential trace elements required for enzyme activity. These combined effects make chitosan an effective, biodegradable, and non-toxic antimicrobial agent widely applied in food preservation, wound healing, and biomedical materials. Figure 4 and Figure 5 represent bioabsorbable active dressings loaded with mesoporous chitosan nanofibers to accelerate the healing of burn wounds and their preparation pathways. Additionally, these amino groups provide reactive sites for chemical modifications and cross-linking reactions, offering extensive opportunities for tailoring chitosan properties for specific packaging applications [27,28,29,30,31,32,33,34,35,36,37,38].

The development of carboxymethyl chitosan–polycaprolactone composite materials through solution blow spinning represents an innovative approach to creating rapid in situ packaging for fruit preservation. Carboxymethyl chitosan, a water-soluble derivative of chitosan obtained through the carboxymethylation of hydroxyl and amino groups, exhibits enhanced solubility across a broader pH range compared to native chitosan while retaining antimicrobial properties. The combination with polycaprolactone, a biodegradable synthetic polyester with excellent mechanical properties and hydrophobicity, creates a composite material that balances the bioactivity and hydrophilicity of carboxymethyl chitosan with the mechanical strength and water resistance of polycaprolactone. Solution blow spinning, a relatively simple and scalable fabrication technique that uses compressed air to draw polymer solutions into fine fibers, enables the rapid production of nonwoven fibrous mats with high surface area and porosity [26,27,28,29]. This technology offers significant advantages for in situ packaging applications where packaging materials are generated on demand at the point of use, reducing storage requirements and enabling customization for specific products.

The application of carboxymethyl chitosan–polycaprolactone fibrous mats for fruit preservation addresses several critical challenges in maintaining post-harvest quality. Fruits undergo rapid quality deterioration after harvest due to continued respiration, moisture loss, microbial contamination, and enzymatic reactions. The high surface area of electrospun or blow-spun fibers facilitates gas exchange, allowing control of the microenvironment around the fruit by modulating oxygen and carbon dioxide concentrations. The antimicrobial properties of carboxymethyl chitosan inhibit fungal and bacterial growth on fruit surfaces, which represents a major cause of post-harvest losses. The mechanical properties and flexibility of the composite mat enable conformable wrapping around irregularly shaped fruits, ensuring intimate contact and uniform protection. The biodegradability of both components addresses environmental concerns associated with conventional plastic fruit packaging and wraps [30,31,32].

The development of pH-responsive cross-linked chitin nanofiber-reinforced chitosan films represents another sophisticated approach to creating intelligent packaging systems. Chitin nanofibers, extracted from crustacean shells or other chitin sources through mechanical or chemical treatments, possess exceptional mechanical properties with tensile strength and a modulus comparable to synthetic high-performance fibers. The incorporation of these nanofibers into chitosan matrices provides significant reinforcement, addressing one of the primary limitations of chitosan films. Cross-linking reactions using agents such as glutaraldehyde, genipin, or citric acid further enhance mechanical properties and water resistance by creating covalent bonds between polymer chains, reducing chain mobility and crystallinity. The pH-responsive functionality is achieved through the incorporation of natural colorimetric indicators, particularly anthocyanins extracted from red cabbage or other colored fruits and vegetables. Anthocyanins are water-soluble pigments belonging to the flavonoid family, composed of an anthocyanidin backbone (flavylium cation) linked to sugars and acyl groups. Their color changes depend strongly on pH, which affects the equilibrium between multiple structural forms. Under acidic conditions (pH < 3), they exist mainly as the flavylium cation, showing a red color. As pH approaches neutrality (pH 4–6), the molecule converts into the quinoidal base, appearing purple to blue, and in alkaline environments (pH 7–9 and pH >9), it further transforms into anionic or chalcone forms, producing greenish or colorless tones. This reversible shift occurs through protonation–deprotonation and hydration reactions affecting the conjugated system of the anthocyanin structure. During food spoilage, microbial activity produces alkaline compounds like ammonia, trimethylamine, and other volatile amines, raising the pH and triggering visible color changes in anthocyanin-containing films. Hence, anthocyanin-based materials serve as natural pH indicators and are increasingly used in intelligent food packaging to provide a visual signal of freshness or spoilage [26].

**Figure 6:**
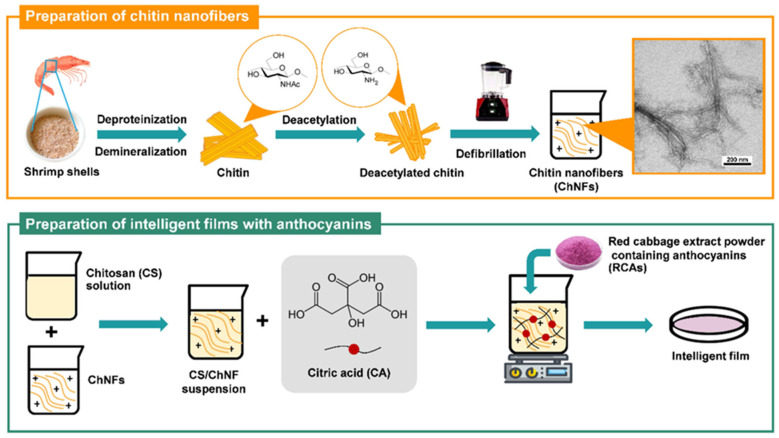
Preparation of chitin nanofibers (ChNFs), with their morphology observed using transmission electron microscopy (TEM), and intelligent films incorporating red cabbage extract powder containing anthocyanins (RCAs) [43].

**Figure 7:**
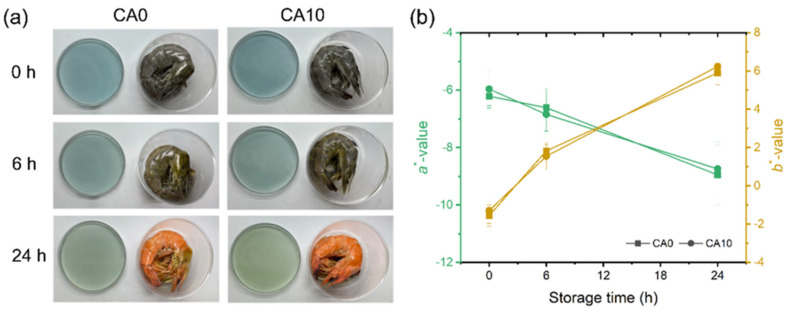
(**a**) Application of the uncross-linked CA0 and cross-linked CA10 films with RCAs to monitor changes in shrimp freshness stored at the room temperature (25 °C ± 3 °C) for 24 h and (**b**) color changes in the uncross-linked CA0 and cross-linked CA10 films for monitoring shrimp freshness [43].

The integration of chitin nanofibers, a cross-linked chitosan matrix, and red cabbage anthocyanins creates a multifunctional packaging material that combines mechanical strength, antimicrobial activity, and real-time freshness monitoring capabilities (Figure 6 and Figure 7). The chitin nanofibers provide structural reinforcement through their high aspect ratio and strong interfacial adhesion with the chitosan matrix, mediated by hydrogen bonding and physical entanglement. Cross-linking enhances water resistance and dimensional stability, critical properties for packaging high-moisture foods. The anthocyanin indicators respond to pH changes associated with food spoilage, providing consumers with visual information about product freshness without requiring sophisticated analytical equipment or destructive testing. The stability of anthocyanins within the cross-linked chitosan–chitin nanofiber matrix represents an important consideration, as anthocyanins are susceptible to degradation through oxidation, light exposure, and pH extremes. The protective environment provided by the polymer matrix, combined with potential stabilizing interactions between anthocyanins and the polysaccharide chains, enhances the longevity and reliability of the color-indicating function [39,40,41,42,43].

Recent research has also explored novel approaches to enhancing chitosan-based packaging through the development of nanochitin–nisin complexes incorporated into multi-component nanofiber systems. Nisin, a bacteriocin produced by Lactococcus lactis, is a widely used natural antimicrobial peptide approved for food applications due to its effectiveness against Gram-positive bacteria and its safety profile. The complexation of nisin with nanochitin creates a controlled-release system where the antimicrobial peptide is protected from degradation and released gradually over time. The incorporation of these complexes into core–shell nanofiber structures composed of pullulan, gelatin, and zein provides additional levels of functionality and control. This sophisticated architecture enables the spatial organization of different components, with antimicrobial complexes potentially located in the core for sustained release or in the shell for immediate action, depending on the desired release profile. Such advanced chitosan-based systems exemplify the evolution toward increasingly sophisticated packaging materials that integrate multiple functional components in precisely engineered architectures [36,37].

### 2.3. Cellulose-Based Packaging Materials

Cellulose, the most abundant biopolymer on Earth, has long been utilized in packaging applications, traditionally in the form of paper and cardboard. Recent advances in nanotechnology and materials processing have enabled the extraction and utilization of cellulose in nanoscale forms, including cellulose nanocrystals, cellulose nanofibers, and bacterial cellulose, which exhibit exceptional mechanical properties, high surface area, and unique structural characteristics that significantly enhance the performance of biopolymer packaging materials.

Bacterial cellulose, produced through fermentation by Acetobacter species, possesses a unique three-dimensional nanofibrillar network structure that confers superior mechanical properties, high water-holding capacity, and excellent biocompatibility compared to plant-derived cellulose. The development of bacterial cellulose–polyvinyl alcohol composite films represents a strategic approach to creating high-barrier active packaging materials for food preservation. Polyvinyl alcohol, a synthetic water-soluble polymer with excellent film-forming properties, mechanical strength, and gas barrier characteristics, complements bacterial cellulose by providing additional matrix support and enhancing processability. The combination creates composite materials that leverage the nanofibrillar reinforcement of bacterial cellulose and the barrier properties of polyvinyl alcohol. The high aspect ratio and strong interfacial adhesion of bacterial cellulose nanofibers within the polyvinyl alcohol matrix result in significant improvements in tensile strength, Young’s modulus, and resistance to crack propagation. The barrier properties against oxygen and water vapor, critical for preventing oxidative deterioration and moisture-related quality changes in packaged foods, are enhanced through the creation of a tortuous path that reduces permeant diffusion rates [38,39,40].

The incorporation of active compounds into bacterial cellulose–polyvinyl alcohol matrices enables the creation of active packaging systems that provide antimicrobial, antioxidant, or other bioactive functionalities. The high surface area and porous structure of bacterial cellulose facilitate the loading and controlled release of bioactive compounds, while the polyvinyl alcohol matrix can be formulated to modulate release kinetics through variations in crystallinity, cross-linking density, and hydrophilicity. Applications of such high-barrier active packaging materials span a wide range of food products, with particular relevance for oxygen-sensitive foods prone to lipid oxidation, such as nuts, oils, and fatty foods, as well as moisture-sensitive products like dried fruits, snacks, and bakery items. The biodegradability of bacterial cellulose combined with the potential for using bio-based polyvinyl alcohol variants addresses sustainability concerns while delivering performance comparable to conventional synthetic packaging materials [39].

The preparation of cellulose nanocrystal biofilms from alternative sources such as coconut coir represents an important development in expanding the raw material base for sustainable packaging while valorizing agricultural waste streams. Coconut coir, the fibrous material between the outer shell and the inner fruit of coconuts, is produced in massive quantities as a by-product of coconut processing and is often underutilized or disposed of as waste. The extraction of cellulose nanocrystals from coconut coir through acid hydrolysis removes amorphous regions of cellulose and lignin, yielding highly crystalline rod-like nanoparticles with exceptional mechanical properties, high surface area, and reactive surface hydroxyl groups. These cellulose nanocrystals can be processed into biofilms through various techniques, including casting, layer-by-layer assembly, or incorporation into polymer matrices as reinforcing nanofillers. The utilization of waste biomass for producing high-value packaging materials exemplifies the principles of circular economy and sustainable resource management, converting agricultural residues into functional products while reducing environmental burden [44,45,46,47,48,49,50,51].

**Figure 8:**
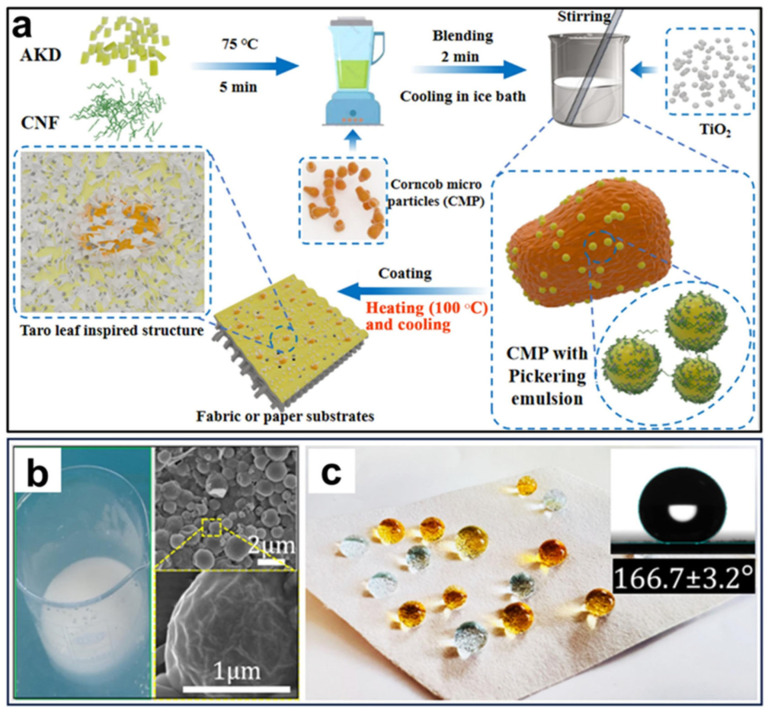
(**a**) Fabrication scheme of A@CCT coating, (**b**) optical and SEM images of as-prepared A@CCT-3 emulsion, and (**c**) optical photo of the colored water droplets on the superhydrophobic paper with A@CCT-3 coating and the corresponding WCA [51].

**Figure 9:**
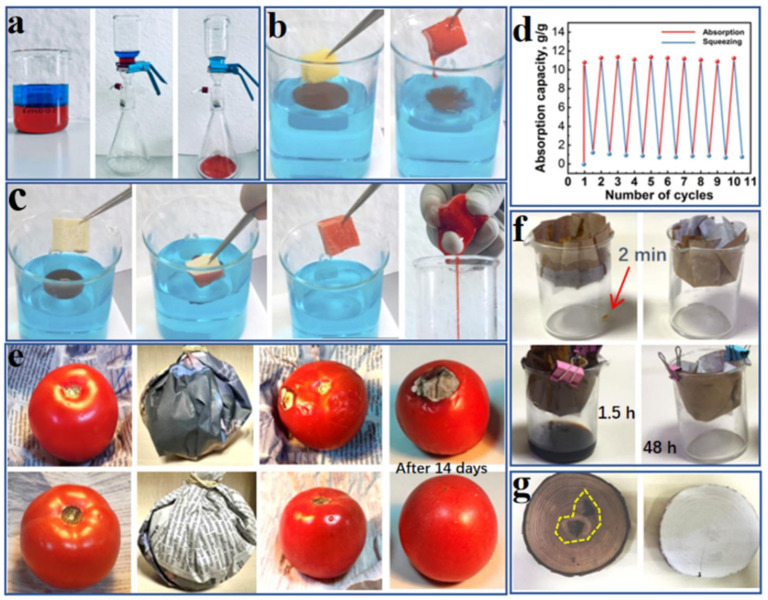
Demonstration of some practical applications of AKD@CCT emulsion. (**a**) Oil/water separation test by filtration, using uncoated (**left**) and coated (**right**) filter paper, (**b**) oil droplet absorption using uncoated sponge, (**c**) oil droplet absorption using AKD@CCT-coated sponge, and (**d**) plot of absorption capacity to cycle, (**e**) anti-rotting test, (**f**) leakage proof test and (**g**) anti-fungal test for uncoated and coated with newspaper [51].

Cellulose nanocrystal films exhibit interesting optical properties, including birefringence and the ability to form chiral nematic structures that produce iridescent colors, potentially enabling their use in anti-counterfeiting applications or decorative packaging (Figure 8 and Figure 9). The mechanical properties of cellulose nanocrystal films, while impressive on a weight-normalized basis, can be limited by brittleness and moisture sensitivity, necessitating strategies for plasticization or combination with other polymers to achieve practical toughness and flexibility. The incorporation of cellulose nanocrystals as reinforcing fillers in biopolymer matrices has proven highly effective in enhancing mechanical properties, with improvements in tensile strength and modulus often observed at relatively low nanocrystal loadings due to percolation effects and the formation of reinforcing networks through hydrogen bonding between nanocrystals and the polymer matrix [22,25,38].

The use of microcrystalline cellulose as a carrier platform for functional compounds, as demonstrated in the starch–pectin–ZIF-67 system discussed previously, illustrates another valuable role for cellulose materials in advanced packaging applications. Microcrystalline cellulose, produced through acid hydrolysis of cellulose fibers to remove amorphous regions while retaining some chain length, exists as micrometer-scale particles with high crystallinity, large surface area, and excellent mechanical properties. These characteristics make microcrystalline cellulose an ideal carrier for loading and dispersing functional compounds, such as antimicrobial agents, antioxidants, or indicator molecules, throughout polymer matrices. The hydrogen bonding capability of cellulose hydroxyl groups facilitates strong interactions with both the functional compounds and the polymer matrix, ensuring stable incorporation and controlled release [38]. The use of cellulose materials in various forms and scales, from nanocrystals to nanofibers to microcrystalline particles to bacterial cellulose networks, demonstrates the versatility of this abundant biopolymer in creating sustainable packaging solutions with tailored properties.

## 3. Other Polysaccharide-Based Systems

Beyond the extensively studied starch, chitosan, and cellulose systems, other polysaccharides, including pectin, pullulan, and dextran, offer unique properties that can be leveraged for specialized packaging applications or to create novel composite materials with synergistic functionalities.

Pectin, as mentioned earlier in the context of starch–pectin composites, is a structural polysaccharide found in plant cell walls, particularly abundant in citrus peels and apple pomace. Pectin consists primarily of galacturonic acid units with varying degrees of esterification, which significantly influences its gelling behavior, mechanical properties, and interactions with other components. High-methoxyl pectin requires acidic conditions and high sugar concentrations for gelation, while low-methoxyl pectin can form gels through ionic cross-linking with divalent cations such as calcium. The ability of pectin to form films and gels, combined with its natural origin, biodegradability, and potential prebiotic effects, makes it attractive for food packaging applications. Pectin films typically exhibit good oxygen barrier properties but high water vapor permeability due to their hydrophilic nature, necessitating strategies for hydrophobization or combination with more hydrophobic materials. The natural antimicrobial properties of pectin, though generally mild, can be enhanced through chemical modifications or incorporation of antimicrobial agents. The use of pectin in composite systems, as demonstrated in the starch–pectin–ZIF-67 formulation, enables the creation of materials that combine pectin’s film-forming ability and biocompatibility with the functional properties of other components [41,42,43].

**Figure 10:**
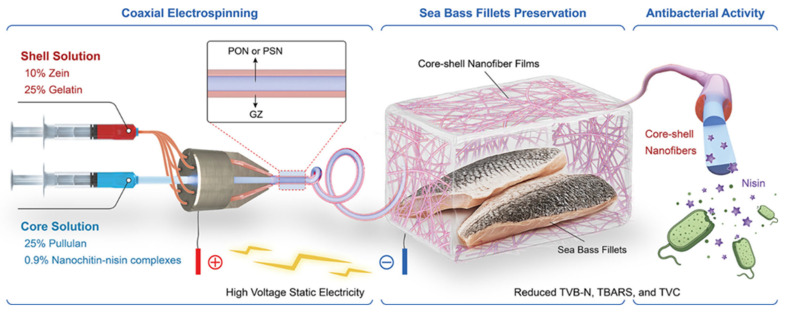
Development of core–shell pullulan–gelatin–zein nanofiber films loaded with nanochitin–nisin complexes for antibacterial food packaging [63].

**Figure 11:**
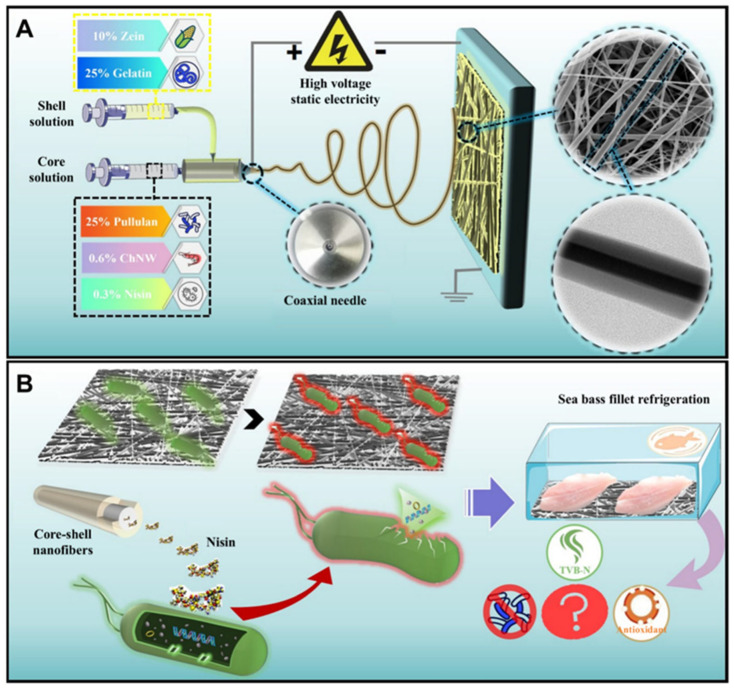
(**A**) Schematic diagram of the preparation of core–shell nanofiber films by coaxial electrospinning technology and (**B**) mechanism explanation of the application of core–shell nanofiber films as antibacterial packaging for seabass preservation [63].

Pullulan, a linear polysaccharide produced by the fungus *Aureobasidium pullulans*, consists of maltotriose units connected by α-1,6-glycosidic bonds, resulting in a unique structure that confers excellent film-forming properties, oxygen barrier characteristics, and edibility. Pullulan films are transparent, colorless, tasteless, and odorless, making them particularly suitable for applications where packaging esthetics and sensory neutrality are important. The oxygen barrier properties of pullulan films rival those of synthetic polymers like polyvinyl alcohol, making pullulan valuable for packaging oxygen-sensitive foods [52,53]. However, similar to other polysaccharides, pullulan exhibits high water vapor permeability and poor moisture resistance, limiting its use in high-humidity environments without modification or combination with hydrophobic materials. Recent research has explored the use of pullulan in multi-component nanofiber systems, as exemplified by the development of core–shell pullulan–gelatin–zein nanofibers loaded with antimicrobial complexes, as shown in Figure 10 and Figure 11. In such systems, pullulan contributes its excellent film-forming properties, oxygen barrier characteristics, and biocompatibility, while gelatin and zein provide complementary functionalities including mechanical properties, water resistance, and protein-based bioactivity. The core–shell architecture enables spatial organization of different components, with pullulan potentially forming part of the shell layer that provides barrier properties and structural integrity [54,55,56,57,58,59,60,61,62,63].

Dextran, another microbially produced polysaccharide consisting of glucose units primarily linked by α-1,6-glycosidic bonds with varying degrees of branching through α-1,3 linkages, has been investigated for food packaging applications, particularly after chemical modifications to enhance its functionality. The esterification of dextran by octenyl succinic anhydride (OSA) represents a chemical modification strategy aimed at introducing hydrophobic groups onto the hydrophilic polysaccharide backbone, thereby improving water resistance, reducing water vapor permeability, and enabling better interaction with hydrophobic food components or active compounds. OSA modification has been extensively used for starches and has been adapted for other polysaccharides, including dextran. The reaction involves the opening of the OSA ring and formation of ester bonds with hydroxyl groups on the dextran chain, resulting in the attachment of octenyl succinic acid moieties that contain both a hydrophobic octenyl tail and an anionic carboxyl group. This amphiphilic character confers emulsifying properties to OSA-modified dextran, enabling its use as a stabilizer for emulsion-based coatings or as a matrix for incorporating lipophilic bioactive compounds [49,50,51,52].

The physicochemical characterization of OSA-modified dextran reveals changes in molecular properties, including increased hydrophobicity, altered solubility characteristics, modified thermal behavior, and enhanced emulsification capacity compared to native dextran. The degree of substitution, defined as the number of OSA groups per glucose unit, critically influences these properties and can be controlled through reaction conditions, including OSA concentration, pH, temperature, and reaction time. Functional property assessments of OSA–dextran demonstrate its potential applications in food packaging as a coating material, emulsifier for active compound delivery, or component in composite films. The biodegradability and biocompatibility of OSA-modified dextran, combined with its regulatory approval for food contact applications, position it as a promising material for sustainable packaging solutions. The ability to tune the hydrophobic–hydrophilic balance through controlled OSA modification enables the design of materials with specific interactions with food surfaces and controlled release characteristics for bioactive compounds [53,54,55,56,57,58].

The exploration of these diverse polysaccharide systems highlights the rich palette of natural materials available for developing sustainable packaging solutions. Each polysaccharide brings unique structural features and properties that can be exploited individually or in combination to create materials tailored for specific applications. The trend toward multi-component composite systems that leverage the complementary properties of different polysaccharides, proteins, and functional additives reflects the increasing sophistication of biopolymer packaging design and the recognition that complex challenges require multifunctional solutions [58,59,60,61,62].

## 4. Protein-Based Packaging Systems

Proteins represent another important class of biopolymers for food packaging applications, offering excellent film-forming properties, nutritional value, and unique functional characteristics derived from their amino acid composition and structural organization. Unlike polysaccharides, proteins contain nitrogen and often sulfur, providing different chemical reactivity and interaction possibilities with food components and active compounds [64,65,66,67].

Gelatin, derived from collagen through partial hydrolysis, is one of the most widely studied proteins for packaging applications due to its excellent film-forming ability, biodegradability, and edibility. Gelatin films exhibit good oxygen barrier properties, particularly at low relative humidity, but suffer from poor water resistance and high water vapor permeability due to the hydrophilic nature of the protein. The mechanical properties of gelatin films, while generally inferior to synthetic polymers, can be enhanced through cross-linking, plasticization, or reinforcement with nanofillers. The incorporation of gelatin into multi-component systems, such as the core–shell pullulan–gelatin–zein nanofibers mentioned earlier, enables the exploitation of gelatin’s film-forming properties and biocompatibility while compensating for its limitations through combination with other materials. Gelatin’s ability to form thermo-reversible gels through the formation of triple–helix structures upon cooling provides processing advantages and enables temperature-responsive behavior in certain applications [68,69,70].

Zein, a prolamin protein derived from corn, offers distinctly different properties compared to gelatin, particularly its hydrophobic character due to high contents of nonpolar amino acids such as leucine, proline, and alanine. Zein films exhibit excellent water resistance, good oxygen barrier properties, and glossy appearance, making them attractive for coating applications and moisture-sensitive food packaging. The hydrophobicity of zein enables better compatibility with lipophilic bioactive compounds and essential oils, facilitating their incorporation into packaging materials. However, zein films tend to be brittle and require plasticization or blending with other polymers to achieve adequate flexibility and toughness. The combination of zein with hydrophilic polymers such as pullulan and gelatin in core–shell nanofiber systems creates materials that balance water resistance with mechanical properties and processability. The core–shell architecture enables strategic placement of zein in layers where water resistance is most critical, while more hydrophilic components contribute to mechanical properties and biocompatibility [71,72,73].

The development of novel core–shell pullulan–gelatin–zein nanofiber films loaded with nanochitin–nisin complexes represents a sophisticated example of protein–biopolymer composite systems designed for antibacterial food packaging. This multi-component system integrates several levels of functionality and structural organization. The core–shell nanofiber architecture, typically produced through coaxial electrospinning, enables the creation of fibers with distinct core and shell compositions, allowing for spatial organization of components with different properties or functions. The high surface area and porosity of nanofiber mats facilitate gas exchange, moisture management, and controlled release of active compounds. The incorporation of nano chitin–nisin complexes provides sustained antimicrobial activity through the gradual release of nisin from the nanochitin carrier. The combination of pullulan, gelatin, and zein in the nanofiber structure creates a material that balances oxygen barrier properties, mechanical strength, water resistance, and biocompatibility. The custom development of such sophisticated systems requires careful optimization of multiple parameters, including polymer solution properties, electrospinning conditions, relative proportions of core and shell materials, and loading levels of active complexes.

The physicochemical properties of these protein–polysaccharide nanocomposite films, including morphology, mechanical properties, barrier characteristics, and antimicrobial activity, depend critically on the successful integration of all components and the maintenance of the nanofiber structure during processing and application. Characterization studies typically reveal that well-designed composite systems exhibit synergistic improvements in properties compared to single-component materials, with mechanical strength enhanced through nanofiber reinforcement, barrier properties improved through multi-component composition, and antimicrobial activity sustained through controlled release mechanisms. The application of such advanced protein-based nanofiber systems for food packaging demonstrates the potential for creating high-performance materials from renewable resources, though challenges remain regarding scalability, cost-effectiveness, and long-term stability under various storage conditions [73,74,75,76,77,78,79].

## 5. Incorporation of Functional Additives and Nanofillers

The enhancement of biopolymer packaging properties through the incorporation of functional additives and nanofillers represents a critical strategy for overcoming the inherent limitations of natural polymers and creating materials with tailored functionalities. This section examines various approaches for incorporating antimicrobial agents, colorimetric indicators, metal–organic frameworks, and other functional components into biopolymer matrices.

### 5.1. Antimicrobial Agents and Essential Oils

**Figure 12:**
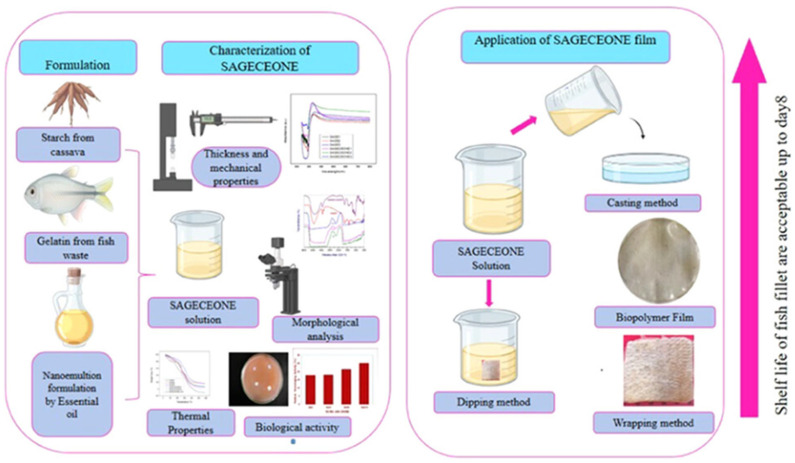
Cinnamon essential oil-infused cassava starch and fish gelatin edible films for extending the shelf life of fish filets [72].

The incorporation of antimicrobial agents into packaging materials enables active packaging systems that inhibit microbial growth on food surfaces, extending shelf life and enhancing safety. Natural antimicrobial compounds, including essential oils, plant extracts, bacteriocins, and organic acids, are particularly attractive due to consumer preference for natural food preservation methods and regulatory considerations favoring generally recognized as safe (GRAS) substances. Essential oils (EOs) exhibit strong antibacterial activity, primarily through their ability to disrupt bacterial cell membranes, interfere with metabolic processes, and induce oxidative stress. Composed mainly of volatile compounds such as terpenes, phenolics, aldehydes, and ketones, EOs are hydrophobic and can penetrate bacterial cell walls and cytoplasmic membranes. This penetration causes membrane destabilization, leading to leakage of ions, ATP, and other vital cell contents. Components like thymol, carvacrol, eugenol, and cinnamaldehyde interact with membrane lipids and proteins, altering permeability and enzyme activity. Additionally, EOs can denature proteins, disrupt electron transport chains, and inhibit DNA and RNA synthesis, ultimately impairing bacterial respiration and energy production. Some EOs also generate reactive oxygen species (ROS), which cause oxidative damage to lipids, proteins, and nucleic acids. The overall antibacterial efficiency depends on both the chemical composition of the oil and the cell wall structure of the target microorganism. Gram-positive bacteria are generally more susceptible due to their less complex outer membranes. Owing to these multifaceted mechanisms, essential oils are widely used as natural antimicrobial agents in food preservation, pharmaceuticals, and packaging materials. Cinnamon essential oil, rich in cinnamaldehyde and other antimicrobial compounds, has been extensively studied for incorporation into biopolymer packaging materials. The development of green packaging solutions incorporating cinnamon essential oil for extending the shelf life of fish filets exemplifies the application of natural antimicrobial agents in active packaging (Figure 12). Fish and seafood products are highly perishable due to their high moisture content, neutral pH, presence of free amino acids, and high levels of polyunsaturated fatty acids susceptible to oxidation. The incorporation of cinnamon essential oil into biopolymer films provides antimicrobial protection against both Gram-positive and Gram-negative bacteria commonly associated with fish spoilage, including *Pseudomonas species, Shewanella putrefaciens*, and various *Enterobacteriaceae*. The volatile nature of essential oil components enables their diffusion from the packaging material into the headspace and onto the food surface, creating an antimicrobial atmosphere that inhibits microbial proliferation [80,81,82,83,84,85].

The challenges associated with incorporating essential oils into hydrophilic biopolymer matrices include poor compatibility due to the hydrophobic nature of essential oils, potential for rapid evaporation and loss of activity, and possible negative impacts on the sensory properties of packaged foods due to strong aromas. Strategies to address these challenges include emulsification of essential oils using surfactants or amphiphilic polymers, encapsulation in nanocarriers such as liposomes or cyclodextrins, and controlled release through matrix design. The evaluation of cinnamon essential oil-containing packaging for fish preservation typically involves monitoring of microbial counts, sensory attributes, chemical indicators of spoilage such as total volatile basic nitrogen and thiobarbituric acid reactive substances, and shelf life extension compared to conventional packaging. Results generally demonstrate significant reductions in microbial growth, delayed onset of off-odors and flavors, and extended shelf life, validating the effectiveness of this natural antimicrobial approach.

Nisin, as mentioned earlier in the context of nanochitin–nisin complexes, represents another important natural antimicrobial agent for food packaging applications. The bacteriocin’s effectiveness against Gram-positive pathogens, including *Listeria monocytogenes* and *Staphylococcus aureus*, makes it particularly valuable for meat and dairy product packaging. The complexation of nisin with carriers such as nanochitin protects the peptide from degradation, controls its release kinetics, and enhances its distribution throughout the polymer matrix. Other antimicrobial peptides, enzymes such as lysozyme, and natural extracts from sources, including green tea, grape seed, and various herbs and spices, have also been investigated for incorporation into biopolymer packaging materials, each offering distinct antimicrobial spectra and compatibility characteristics [86,87,88,89].

### 5.2. Colorimetric Indicators and Intelligent Packaging Components

The integration of colorimetric indicators into packaging materials enables the development of intelligent packaging systems that provide real-time information about food quality, freshness, or storage conditions through visible color changes. This functionality addresses consumer demand for transparent information about food quality and reduces food waste by enabling more accurate assessment of product freshness compared to relying solely on printed expiration dates.

Anthocyanins, as discussed in the context of pH-responsive chitosan films, represent the most extensively studied class of natural colorimetric indicators for intelligent food packaging. These water-soluble pigments, responsible for red, purple, and blue colors in many fruits, vegetables, and flowers, exhibit pH-dependent color changes due to structural transformations of the anthocyanin molecule. The enhanced stability of anthocyanins through encapsulation or complexation represents an important consideration for their practical application in packaging materials. Recent research has demonstrated that cyclodextrin metal–organic framework hybrid systems can significantly enhance anthocyanin stability while maintaining colorimetric responsiveness. Cyclodextrins are cyclic oligosaccharides with a hydrophobic cavity that can accommodate guest molecules through inclusion complexation, providing protection from degradation factors such as oxygen, light, and pH extremes. Metal–organic frameworks, crystalline porous materials composed of metal ions or clusters coordinated to organic ligands, offer high surface area, tunable pore sizes, and the ability to interact with guest molecules through various mechanisms, including coordination bonding, hydrogen bonding, and π-π interactions [38].

**Figure 13:**
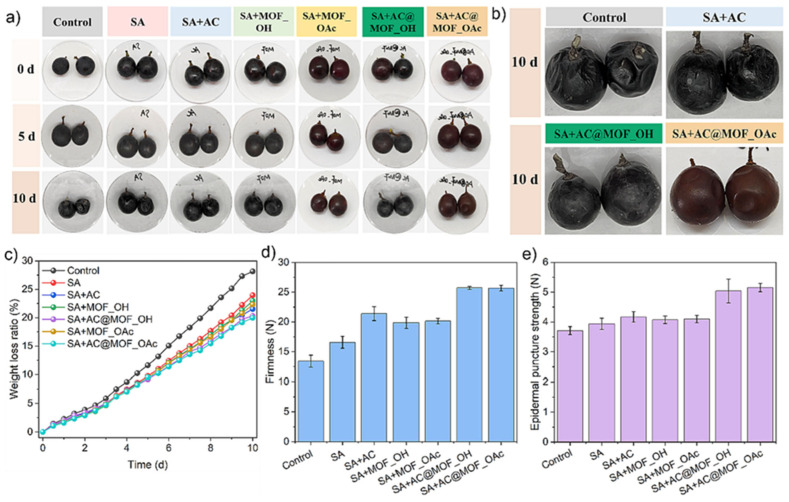
(**a**) Morphological photos; (**b**) the enlarged photos of grapes coated by different films during the 10 days of storage and the corresponding grape preservation index, including (**c**) weight loss ratio, (**d**) firmness, and (**e**) epidermal puncture strength [85].

The encapsulation mechanism of anthocyanins in cyclodextrin metal–organic framework systems involves the formation of inclusion complexes between anthocyanins and cyclodextrin cavities, followed by incorporation of these complexes into the porous structure of metal–organic frameworks (Figure 13). This dual-level protection significantly enhances anthocyanin’s stability against thermal degradation, oxidation, and light-induced bleaching, while maintaining the pH-responsive color change functionality essential for freshness indication. The application of such stabilized anthocyanin systems as protecting agents for grape preservation demonstrates the dual functionality of these materials, providing both antioxidant protection through anthocyanin’s radical scavenging activity and visual indication of storage conditions or quality changes. Grapes, being non-climacteric fruits with high water content and delicate skin, are susceptible to moisture loss, microbial contamination, and oxidative deterioration during storage and transportation. The incorporation of stabilized anthocyanins into packaging materials or as direct coatings on grape surfaces can extend shelf life through antioxidant activity while providing consumers with visual feedback about product quality through color changes associated with pH shifts or oxidative conditions [90,91,92,93].

The development of pH-responsive packaging materials requires careful consideration of the pH range relevant to the specific food application, the sensitivity and reversibility of the indicator response, and the stability of the indicator system under storage conditions. For protein-rich foods such as meat and fish, spoilage typically involves the production of alkaline compounds through microbial metabolism and protein degradation, resulting in pH increases from initial values around 5.5–6.5 to values above 7.0–8.0 in spoiled products. Anthocyanin indicators that exhibit clear color transitions in this pH range, such as those from red cabbage, which transition from pink-red to purple to blue-green with increasing pH, are particularly suitable for meat and fish packaging. For other food types, different pH ranges and indicator systems may be more appropriate, requiring customization of the intelligent packaging approach to the specific application.

### 5.3. Metal–Organic Frameworks and Advanced Nanofillers

The incorporation of metal–organic frameworks into biopolymer packaging materials represents an emerging frontier in creating multifunctional systems that combine antimicrobial activity, catalytic properties, gas adsorption capabilities, and sensing functionalities. Metal–organic frameworks’ high surface area, tunable pore structures, and diverse metal–ligand combinations enable the design of materials with specific interactions with food components, spoilage compounds, or environmental gases. The use of zeolitic imidazolate framework-67 (ZIF-67) in starch–pectin composite films, as discussed earlier, exemplifies the potential of metal–organic frameworks in food packaging. ZIF-67, composed of cobalt ions coordinated to 2-methylimidazolate ligands in a zeolitic topology, exhibits antimicrobial activity through cobalt ion release and demonstrates a colorimetric response to nitrogen-containing compounds through coordination interactions with the metal centers. The purple color of ZIF-67 changes upon exposure to ammonia or volatile amines, providing a visual indication of food spoilage [38].

The loading of metal–organic frameworks onto carrier materials such as microcrystalline cellulose addresses challenges related to aggregation and poor dispersion of nanoparticles in polymer matrices. The high surface area and hydroxyl-rich surface of microcrystalline cellulose facilitate the anchoring of metal–organic framework particles through hydrogen bonding and coordination interactions, creating a composite filler that combines the mechanical reinforcement of cellulose with the functional properties of metal–organic frameworks. This hierarchical structuring approach enables the incorporation of relatively high loadings of functional nanoparticles while maintaining good dispersion and interfacial adhesion with the polymer matrix, critical factors for achieving uniform properties and avoiding agglomeration-related defects.

Other advanced nanofillers, including silver nanoparticles, zinc oxide nanoparticles, titanium dioxide nanoparticles, and carbon-based nanomaterials, have been investigated for incorporation into biopolymer packaging materials, each offering distinct functionalities. Silver nanoparticles provide broad-spectrum antimicrobial activity through multiple mechanisms, including silver ion release, reactive oxygen species generation, and direct interaction with bacterial cell membranes. Zinc oxide nanoparticles offer antimicrobial properties combined with UV-blocking capabilities, protecting both the packaged food and the packaging material from photodegradation. Titanium dioxide nanoparticles can provide photocatalytic self-cleaning properties and UV protection. Carbon-based nanomaterials, including graphene, graphene oxide, and carbon nanotubes, offer exceptional mechanical reinforcement, electrical conductivity for potential sensing applications, and barrier property enhancement through the creation of tortuous diffusion paths. The selection of appropriate nanofillers depends on the specific application requirements, food safety considerations, regulatory status, and cost-effectiveness, with natural and bio-based nanofillers generally preferred for sustainable packaging solutions [94,95,96,97,98].

## 6. Advanced Processing and Fabrication Technologies

The development of sophisticated biopolymer packaging materials with tailored structures and properties requires advanced processing and fabrication technologies beyond conventional film casting or extrusion methods. This section examines emerging technologies, including electrospinning, solution blow spinning, cryogelation, and various modification techniques, which enable the creation of nanostructured, responsive, and multifunctional packaging materials.

### 6.1. Electrospinning and Solution Blow Spinning

Electrospinning, a versatile technique for producing continuous fibers with diameters ranging from nanometers to micrometers, has attracted significant attention for creating high-surface-area packaging materials with controlled porosity and nanostructures. The process involves applying a high voltage to a polymer solution or melt, causing the formation of a charged jet that undergoes stretching and solvent evaporation, resulting in the deposition of fine fibers on a collector. The high surface area-to-volume ratio of electrospun nanofibers facilitates enhanced contact with food surfaces, efficient gas exchange, and controlled release of active compounds. The porous structure of electrospun mats enables moisture management and breathability while maintaining barrier properties against larger contaminants. The ability to produce core–shell, hollow, or porous fibers through modifications of the electrospinning setup and process parameters enables sophisticated control over material architecture and functionality [72].

The development of core–shell nanofibers through coaxial electrospinning represents a particularly powerful approach for creating materials with the spatial organization of different components. In this technique, two polymer solutions are simultaneously electrospun through concentric nozzles, resulting in fibers with distinct core and shell layers. This architecture enables the encapsulation of active compounds, sensitive materials, or incompatible components in the core, while the shell provides mechanical support, barrier properties, or controlled release functionality. The core–shell pullulan–gelatin–zein nanofibers loaded with nanochitin–nisin complexes exemplify this approach, with the potential for locating antimicrobial complexes in specific layers to achieve desired release profiles. The customization of core–shell nanofiber properties requires optimization of multiple parameters, including solution properties (concentration, viscosity, and conductivity), process parameters (voltage, flow rate, and needle-to-collector distance), and environmental conditions (temperature and humidity), making it a complex but highly versatile fabrication method.

Solution blow spinning represents an alternative to electrospinning that offers certain practical advantages, including simpler equipment requirements, higher production rates, and elimination of the need for high voltage. In solution blow spinning, the polymer solution is extruded through a nozzle surrounded by a high-velocity gas stream, typically compressed air, which draws the solution into fine fibers through aerodynamic forces. The fibers are collected on a substrate to form nonwoven mats similar to electrospun materials. The rapid production capability of solution blow spinning makes it particularly attractive for industrial-scale manufacturing and in situ packaging applications where materials are generated on-demand. The development of carboxymethyl chitosan–polycaprolactone packaging through solution blow spinning demonstrates the applicability of this technique for creating functional biopolymer composites. The ability to rapidly produce fibrous packaging materials at the point of use enables customization for specific products, reduces storage requirements for packaging materials, and potentially lowers the costs associated with transportation and inventory management.

Both electrospinning and solution blow spinning enable the incorporation of nanoparticles, active compounds, and functional additives into fibers during the spinning process, creating composite materials with distributed functionality. The alignment of fibers can be controlled through collector design, enabling the creation of materials with anisotropic properties, such as directional mechanical strength or controlled permeability. The post-processing of electrospun or blow-spun mats through cross-linking, heat treatment, or solvent vapor exposure can further modify properties, including the mechanical strength, water resistance, and fiber bonding, tailoring the materials for specific applications.

### 6.2. Cryogelation and Three-Dimensional Network Formation

Cryogelation, the process of forming gels at sub-zero temperatures, produces materials with unique macroporous structures that differ fundamentally from conventional hydrogels formed at ambient temperatures. During cryogelation, the aqueous solution containing monomers or polymers and cross-linking agents is frozen, leading to the formation of ice crystals that act as porogens. The unfrozen liquid microphase between the ice crystals becomes concentrated in solutes, facilitating polymerization or cross-linking reactions. Upon thawing, the ice crystals melt, leaving behind interconnected macropores with sizes typically ranging from tens to hundreds of micrometers. This macroporous structure confers several advantageous properties, including excellent mechanical strength, shape recovery after compression, rapid swelling and deswelling kinetics, and the ability to withstand freeze–thaw cycles.

The development of antimicrobial starch-based cryogels for food packaging applications leverages these unique properties to create materials that combine structural functionality with bioactive properties. The macroporous architecture facilitates the loading of antimicrobial agents and their controlled release, while the mechanical properties provide cushioning and protection for packaged foods. The interconnected pore structure enables fluid transport and gas exchange, which are important for maintaining appropriate microenvironments around food products. The ability to control pore size and structure through the manipulation of freezing conditions, polymer concentration, and cross-linking density enables tailoring of cryogel properties for specific applications. The dual-active nature of these cryogels, providing both physical protection and antimicrobial activity, represents an integrated approach to food packaging that addresses multiple preservation requirements simultaneously.

Cryogels can be produced in various forms, including monoliths, sheets, or particles, depending on the mold geometry and processing conditions. The incorporation of nanofillers, active compounds, or other functional additives into the pre-gel solution enables the creation of composite cryogels with enhanced or additional properties [15,26,38]. The biocompatibility and biodegradability of biopolymer-based cryogels, combined with their mechanical robustness and functional capabilities, position them as promising materials for sustainable active packaging, though challenges remain regarding scalability, cost-effectiveness, and integration into existing packaging workflows.

### 6.3. Chemical Modifications and Cross-Linking Strategies

Chemical modifications of biopolymers represent essential strategies for tailoring their properties to meet specific packaging requirements, particularly regarding water resistance, mechanical strength, and functional group availability. Various modification approaches, including esterification, etherification, grafting, oxidation, and cross-linking, have been applied to polysaccharides and proteins to enhance their performance in packaging applications.

Esterification reactions, such as the modification of dextran with octenyl succinic anhydride discussed earlier, introduce hydrophobic groups onto hydrophilic polymer backbones, reducing water sensitivity and enabling better interaction with lipophilic compounds. The degree of substitution achieved through esterification critically influences the resulting properties, with higher substitution generally leading to increased hydrophobicity but potentially affecting biodegradability and biocompatibility. The esterification of starch with various anhydrides or acids has been extensively studied for creating water-resistant films and coatings, with acetylation being one of the most common modifications. The physicochemical characterization of esterified biopolymers typically involves the determination of the degree of substitution through titration or spectroscopic methods, assessment of solubility and swelling behavior, thermal analysis to evaluate glass transition and melting behavior, and functional property evaluation including water contact angle, moisture uptake, and mechanical properties [26].

Cross-linking represents another critical modification strategy that creates covalent bonds between polymer chains, reducing chain mobility, decreasing crystallinity, and enhancing mechanical properties and water resistance. Chemical cross-linking agents, including glutaraldehyde, genipin, citric acid, and various multifunctional epoxides or isocyanates, have been employed for biopolymer modification. Glutaraldehyde, while highly effective for cross-linking proteins and polysaccharides containing amino groups, raises toxicity concerns that limit its use in food contact applications. Genipin, a natural cross-linking agent derived from gardenia fruits, offers a safer alternative with lower toxicity and the ability to cross-link through reactions with amino groups. Citric acid can cross-link hydroxyl-containing polymers through esterification reactions under appropriate conditions, offering a food-safe and cost-effective option. The optimization of cross-linking conditions, including agent concentration, pH, temperature, and reaction time, is essential for achieving the desired property improvements while maintaining material safety and biodegradability.

Physical cross-linking through mechanisms such as ionic interactions, hydrogen bonding, or crystallite formation offers alternatives to chemical cross-linking that avoid the use of potentially toxic cross-linking agents. The cross-linking of low-methoxyl pectin with calcium ions, the formation of polyelectrolyte complexes between oppositely charged polysaccharides, and the development of crystalline regions through annealing or controlled drying represent examples of physical cross-linking approaches. These methods generally result in reversible or environmentally responsive cross-links that can provide stimuli-responsive behavior, enabling the design of smart packaging materials that respond to temperature, pH, or ionic strength changes [38,43].

## 7. Applications in Food Preservation

The practical validation of biopolymer packaging materials requires rigorous testing with actual food products under realistic storage conditions, evaluating their effectiveness in extending shelf life, maintaining quality, and ensuring safety. This section examines applications of advanced biopolymer packaging systems for preserving various food categories including meat, fish, fruits, and other perishables.

### 7.1. Meat and Poultry Packaging

Meat and poultry products present significant packaging challenges due to their high protein and moisture content, neutral pH, and susceptibility to microbial contamination, lipid oxidation, and color deterioration. The application of multifunctional starch–pectin composite films engineered with ZIF-67-loaded microcrystalline cellulose for pork preservation demonstrates the potential of intelligent active packaging to address these challenges. Fresh pork undergoes rapid quality degradation through multiple mechanisms, including microbial growth, particularly by psychrotrophic bacteria that proliferate at refrigeration temperatures, oxidation of lipids leading to rancidity and off-flavors, and protein degradation, producing volatile basic nitrogen compounds including ammonia, trimethylamine, and other amines. The antimicrobial activity provided by ZIF-67 inhibits bacterial growth, extending the lag phase and reducing the growth rate during the exponential phase, thereby delaying the point at which microbial counts exceed acceptable limits. The barrier properties of the starch–pectin–cellulose matrix reduce oxygen permeation, slowing lipid oxidation and helping maintain color stability [38]. The colorimetric indication provided by ZIF-67’s response to volatile nitrogen compounds enables real-time monitoring of freshness, with color changes providing a visible warning of quality deterioration. Shelf life studies typically involve the storage of packaged pork samples at refrigeration temperatures with periodic evaluation of microbial counts, pH, total volatile basic nitrogen, thiobarbituric acid reactive substances as indicators of lipid oxidation, color parameters, and sensory attributes. Results from such studies generally demonstrate significant extensions in shelf life, often by several days compared to conventional packaging, along with better maintenance of color, lower accumulation of oxidation products, and delayed development of off-odors. The practical implementation of such intelligent active packaging for meat products requires consideration of factors including packaging format (trays with film overwrap, vacuum bags, and modified atmosphere packaging), compatibility with existing processing and distribution infrastructure, cost-effectiveness compared to conventional materials, and consumer acceptance of the color-changing functionality [96,97,98,99,100].

The pH-responsive chitosan–chitin nanofiber films with anthocyanin indicators represent another approach to intelligent meat packaging, with the pH-responsive color change providing freshness indication through a different mechanism. The accumulation of volatile basic nitrogen compounds during meat spoilage increases pH, triggering anthocyanin color changes from red through purple to blue-green. The antimicrobial activity of chitosan provides additional preservation benefits, while the mechanical reinforcement from chitin nanofibers ensures adequate strength for handling and protection. The cross-linking of the chitosan matrix enhances water resistance, critical for packaging high-moisture meat products.

### 7.2. Seafood Packaging

Fish and seafood products are among the most perishable food items, with shelf life often limited to only a few days even under refrigeration due to high moisture content, high levels of free amino acids and trimethylamine oxide that support microbial growth, and polyunsaturated fatty acids susceptible to oxidation. The development of green packaging solutions incorporating cinnamon essential oil for fish filet preservation addresses these challenges through natural antimicrobial and antioxidant activities. Cinnamon essential oil’s primary component, cinnamaldehyde, exhibits broad-spectrum antimicrobial activity against spoilage bacteria including *Pseudomonas* species, which dominate the spoilage flora of aerobically stored fish, and against pathogenic bacteria such as *Listeria monocytogenes*. The antioxidant compounds in cinnamon essential oil, including eugenol and various phenolic compounds, help prevent lipid oxidation, maintaining sensory quality and nutritional value [85].

The incorporation of cinnamon essential oil into biopolymer films or coatings for fish packaging requires careful optimization to achieve effective antimicrobial concentrations while avoiding excessive sensory impact from the strong cinnamon aroma. Encapsulation strategies using cyclodextrins, liposomes, or nanoemulsions can help control release rates and reduce initial aroma intensity while maintaining antimicrobial efficacy over time. Shelf life studies with fish filets packaged in cinnamon essential oil-containing materials typically demonstrate significant reductions in total viable counts and psychrotrophic bacterial counts, delayed onset of fishy odors associated with trimethylamine production, lower accumulation of total volatile basic nitrogen, and extended sensory acceptability. The extension of shelf life by several days represents substantial economic value given the high cost of fish products and the significant losses currently occurring due to spoilage [43,63,73].

**Figure 14:**
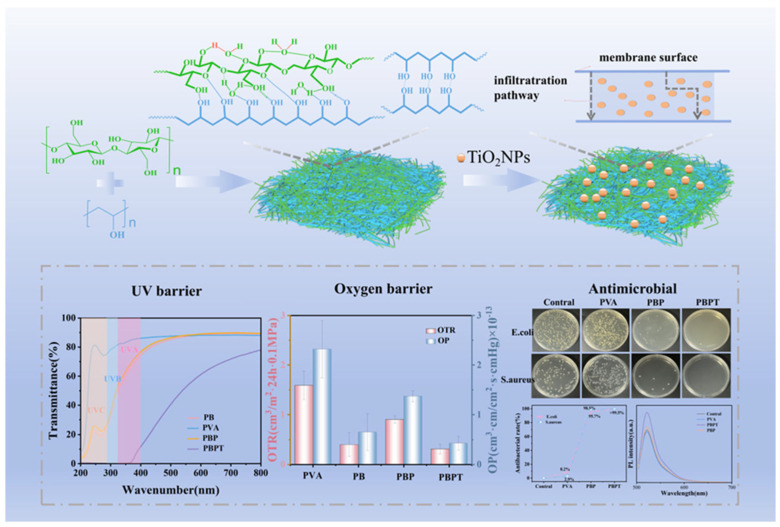
Synthesis and properties of cellulose/polyvinyl alcohol-based active packaging material for food preservation [92].

The high-barrier bacterial cellulose–polyvinyl alcohol active packaging materials offer another approach to seafood preservation, with enhanced oxygen barrier properties helping prevent lipid oxidation, a major quality issue in fatty fish species (Figure 14). The incorporation of antimicrobial agents into these high-barrier matrices provides dual protection against both oxidative and microbial deterioration. The mechanical strength and water resistance of bacterial cellulose–polyvinyl alcohol composites enable their use in direct contact with high-moisture seafood products without excessive swelling or loss of integrity.

**Figure 15:**
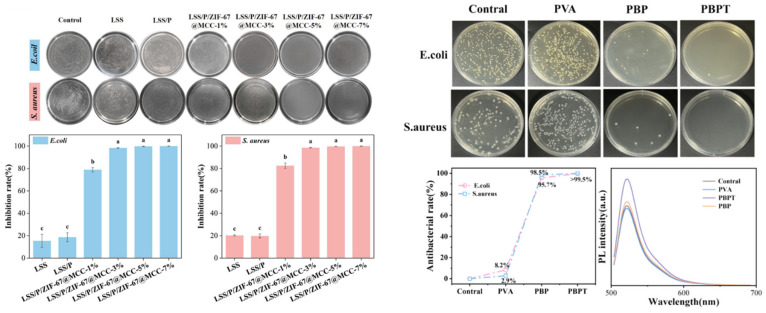
Antimicrobial performance of bio-based films. Photographs of the plate colony-counting method used to visualize surviving bacteria; bacterial growth inhibition rates showing the comparative efficacy of the different film formulations; and quantitative analysis of reactive oxygen species (ROS) production under irradiation, illustrating the mechanism of enhanced antibacterial activity [26,92].

Recent research on bio-based packaging materials has demonstrated significant antibacterial activity through the measurement of inhibition zones and other quantitative metrics (Figure 15). For instance, mesoporous chitosan nanofibers loaded with norfloxacin and coated with phenylboronic acid have shown clear antimicrobial activity against Gram-positive, Gram-negative, and fungal strains. Similarly, edible films composed of cassava starch and fish gelatin infused with cinnamon essential oil nanoemulsions exhibited excellent antibacterial performance. Starch-based cryogels and hydrogels also proved effective against Gram-positive bacterial strains, particularly after being loaded with antimicrobial agents like diacetyl or mint essential oil. These findings highlight the potential of natural biopolymers to act as active carriers for inhibiting microbial contamination in food and medical applications. Beyond traditional starch and chitosan, advanced composite materials have been developed with enhanced antimicrobial properties for food preservation. A loquat seed starch/pectin composite film engineered with ZIF-67-loaded microcrystalline cellulose was found to exhibit superior antibacterial activity, contributing to the preservation of pork freshness for up to 8 days. Additionally, high-barrier films based on bacterial cellulose and polyvinyl alcohol (PVA) incorporating TiO_2_ achieved antimicrobial rates exceeding 99% against common pathogens such as *E. coli* and Staphylococcus aureus. Other innovations include core–shell pullulan–gelatin–zein nanofiber films that utilize nanochitin–nisin complexes to significantly reduce total viable counts and spoilage markers in refrigerated fish filets. These diverse research findings underscore a growing trend toward multifunctional, biodegradable packaging solutions that leverage natural compounds to ensure food safety and extend shelf life [26,92].

### 7.3. Fruit and Vegetable Packaging

Fruits and vegetables continue to respire after harvest, consuming oxygen and producing carbon dioxide, water vapor, and ethylene in the case of climacteric fruits. The management of this respiration process through packaging design represents a critical factor in maintaining post-harvest quality. The development of carboxymethyl chitosan–polycaprolactone rapid in situ packaging through solution blow spinning for fruit preservation demonstrates an innovative approach that combines antimicrobial activity with breathability and on-demand production. The high surface area and porosity of blow-spun fibrous mats enable gas exchange, allowing control of the oxygen and carbon dioxide concentrations around the fruit to slow respiration without inducing anaerobic conditions that lead to off-flavor developments. The antimicrobial properties of carboxymethyl chitosan inhibit fungal and bacterial growth on fruit surfaces, which represents a major cause of post-harvest losses, particularly for fruits with damaged skin or high sugar content that supports microbial proliferation [51,85].

The application of enhanced anthocyanin stability systems using cyclodextrin metal–organic frameworks as protecting agents for grape preservation illustrates the dual functionality of providing both antioxidant protection and potential quality indication. Grapes are susceptible to moisture loss, leading to shriveling, microbial contamination, particularly by fungi such as *Botrytis cinerea*, and oxidative deterioration affecting color and flavor. The antioxidant activity of anthocyanins helps maintain quality by scavenging reactive oxygen species and preventing oxidative damage to pigments, vitamins, and other quality attributes. The stabilization of anthocyanins through cyclodextrin and metal–organic framework encapsulation ensures sustained antioxidant activity throughout the storage period. The potential for using the same anthocyanin systems as colorimetric indicators provides additional value through quality monitoring functionality [98].

**Figure 16:**
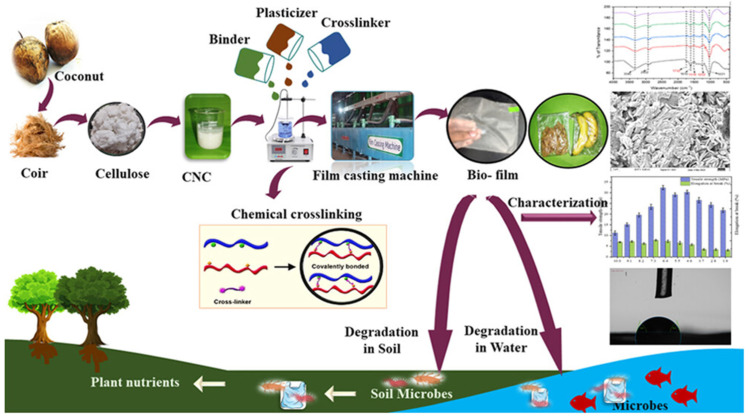
Schematic representation of preparation of cellulose nanocrystals biofilm from coconut coir [98].

**Figure 17:**
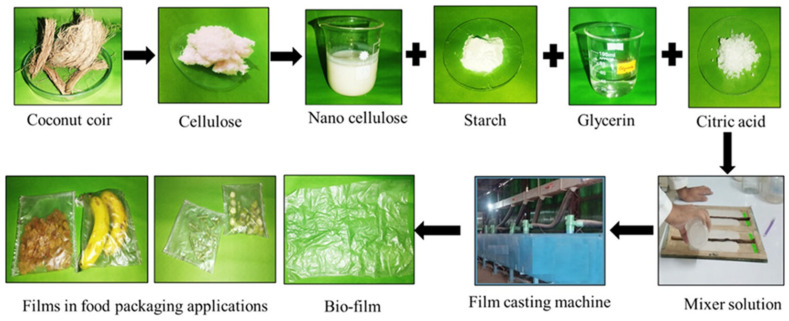
Diagrammatic depiction of the fabrication of the biofilm using CNC, binder (starch), plasticizer (glycerol), and cross-linker (citric acid) as food packaging materials [98].

The cellulose nanocrystal biofilms derived from coconut coir and other agricultural waste sources offer sustainable options for fruit and vegetable packaging, particularly as components of composite materials or coatings (Figure 16 and Figure 17). The mechanical reinforcement provided by cellulose nanocrystals enhances the performance of biopolymer films, while their derivation from waste biomass adds value to agricultural residues and reduces environmental impact. The application of biopolymer coatings containing cellulose nanocrystals directly onto fruit surfaces can reduce moisture loss, provide a barrier against microbial contamination, and deliver active compounds for preservation. Table 1 represents the summary of modified biopolymer materials for active and intelligent food packaging applications.

## 8. Challenges and Limitations

Despite significant advances in biopolymer packaging development, numerous challenges remain that must be addressed to enable widespread commercial adoption and replacement of conventional synthetic packaging materials. This section critically examines the major limitations and obstacles facing the field.

### 8.1. Mechanical and Barrier Property Limitations

The mechanical properties of most biopolymer films, including tensile strength, elongation at break, and puncture resistance, generally remain inferior to conventional synthetic polymers such as polyethylene, polypropylene, and polyethylene terephthalate. This limitation restricts the application of biopolymer materials to situations where mechanical demands are modest or where the materials can be used in composite structures with synthetic polymers providing mechanical support. The brittleness of many biopolymer films, particularly at low moisture contents, poses challenges for processing, handling, and consumer use. Plasticizers such as glycerol, sorbitol, or polyethylene glycol are commonly added to improve flexibility, but these hydrophilic plasticizers can increase water vapor permeability and reduce mechanical strength, requiring careful optimization of plasticizer type and concentration.

Water vapor permeability represents a critical limitation for many polysaccharide- and protein-based films due to their hydrophilic nature and the presence of hydroxyl, amino, and carboxyl groups that interact strongly with water molecules. High water vapor permeability limits the application of these materials for packaging moisture-sensitive foods or for use in high-humidity environments. Various strategies have been employed to reduce water vapor permeability, including chemical modifications to increase hydrophobicity, incorporation of hydrophobic nanofillers or lipids, creation of multilayer structures with alternating hydrophilic and hydrophobic layers, and cross-linking to reduce free volume and chain mobility. However, achieving water vapor barrier properties comparable to synthetic polymers while maintaining biodegradability and processability remains challenging.

Oxygen barrier properties of many biopolymers, particularly at low relative humidity, can be excellent and sometimes superior to synthetic polymers. However, the moisture sensitivity of these barrier properties represents a significant limitation, with oxygen permeability often increasing dramatically at high relative humidity due to the plasticization effects of absorbed water. This moisture dependence limits the application of biopolymer materials for packaging products with high water activity or for use in humid environments without protective layers or modifications to reduce moisture sensitivity.

### 8.2. Scalability and Processing Challenges

The translation of laboratory-scale biopolymer packaging materials to industrial-scale production faces numerous technical and economic challenges. Many advanced fabrication techniques including electrospinning, coaxial electrospinning, and cryogelation, while enabling sophisticated material structures and properties, have limited throughput and require specialized equipment that may not be compatible with existing packaging production infrastructure. The solution blow spinning technique offers better scalability compared to electrospinning, but industrial implementation still requires the development of appropriate equipment and process control systems. The processing of biopolymer materials using conventional packaging equipment, such as extrusion lines, blown film lines, or thermoforming machines, often encounters difficulties due to the thermal sensitivity of natural polymers, their tendency to degrade at processing temperatures, and their different rheological behavior compared to synthetic polymers.

The incorporation of active compounds, nanofillers, and functional additives into biopolymer matrices at the industrial scale presents challenges related to achieving uniform dispersion, maintaining stability during processing, and ensuring reproducible properties across large production batches. The use of organic solvents in many laboratory-scale processes raises environmental and safety concerns for industrial implementation, necessitating the development of water-based or solvent-free processing methods. The drying of biopolymer films, particularly those with high initial moisture content, requires careful control to prevent defects such as cracking, warping, or non-uniform thickness, and represents a time-consuming and energy-intensive step that impacts production economics.

The fabrication methods detailed in recent findings are specifically chosen for their compatibility with existing industrial infrastructure. Techniques such as solvent casting, electrospinning, and pilot-scale reactive twin-screw extrusion are already established in the global packaging and textile sectors, facilitating a smoother transition to mass production. The development of waterborne, non-fluorinated coatings and the use of mild cross-linking agents like citric acid also minimize the need for hazardous solvent handling and specialized safety equipment. By demonstrating that these films can maintain high tensile strength and gas barrier properties under industrial-like conditions, this research paves the way for a viable, large-scale replacement for non-biodegradable plastics in the global supply chain.

### 8.3. Stability and Shelf Life of Packaging Materials

The stability of biopolymer packaging materials during storage before use represents an important consideration that is often inadequately addressed in research studies focused on food preservation performance. Many biopolymer films are moisture-sensitive and can undergo changes in mechanical properties, dimensions, and functionality when stored under varying humidity conditions. The migration of plasticizers, active compounds, or other additives over time can affect material properties and potentially lead to non-compliance with food contact regulations. The stability of colorimetric indicators such as anthocyanins within packaging materials during extended storage requires careful evaluation, as degradation of indicator compounds would compromise the intelligent packaging functionality.

The biodegradability of biopolymer materials, while advantageous for end-of-life disposal, can potentially lead to premature degradation during storage or use if not properly controlled. The susceptibility of some biopolymers to microbial degradation necessitates careful storage conditions and may require the incorporation of preservatives or antimicrobial agents into the packaging material itself. The photodegradation of certain biopolymers and active compounds under light exposure requires consideration of packaging and storage conditions for the packaging materials themselves. These stability considerations impact the shelf life of packaged products and the practical logistics of packaging material distribution and inventory management.

### 8.4. Cost and Economic Viability

The cost of biopolymer packaging materials remains significantly higher than conventional synthetic polymers in most cases, representing a major barrier to commercial adoption. The prices of purified biopolymers such as chitosan, bacterial cellulose, pullulan, and modified starches typically exceed those of commodity plastics by factors ranging from several times to orders of magnitude. The incorporation of functional additives, including essential oils, natural extracts, nanoparticles, and metal–organic frameworks, further increases material costs. The processing costs associated with advanced fabrication techniques and the potential need for specialized equipment add to the economic burden. While the environmental benefits of biopolymer packaging and the potential for reducing food waste through improved preservation provide value propositions that may justify higher costs in some applications, price competitiveness with conventional materials remains essential for widespread adoption.

The development of biopolymer packaging materials from waste biomass, as exemplified by cellulose nanocrystals from coconut coir, represents one strategy for improving economic viability by valorizing low-cost feedstocks. The use of abundant and inexpensive polysaccharides such as starch as the base material, with property enhancement through modest amounts of more expensive functional additives, offers another approach to cost management. The potential for reduced disposal costs due to biodegradability and compostability may partially offset higher material costs, though the infrastructure for industrial composting of biopolymer packaging remains limited in many regions. Life cycle cost analyses that consider not only material and processing costs but also environmental externalities, waste management costs, and potential revenue from reduced food waste are needed to comprehensively assess the economic viability of biopolymer packaging solutions.

The economic feasibility of these active packaging solutions is largely driven by the use of low-cost, sustainable raw materials. By valorizing agricultural and industrial waste—such as starch biomass, fish gelatin from processing leftovers, and cellulose from coconut coir—researchers have significantly reduced the “green premium” typically associated with bioplastics. Furthermore, the integration of natural antimicrobial agents like cinnamon essential oil and anthocyanins from red cabbage or grapes offers a cost-effective alternative to expensive synthetic preservatives. These materials provide a dual economic benefit: they utilize inexpensive feedstocks while simultaneously reducing financial losses for retailers by extending the shelf life of high-value perishables, such as fish and pork, through active preservation.

### 8.5. Regulatory and Safety Considerations

The regulatory approval of biopolymer packaging materials for food contact applications requires the demonstration of safety through migration testing, toxicological evaluation of materials and additives, and compliance with food contact regulations in relevant jurisdictions. The incorporation of nanomaterials, metal–organic frameworks, essential oils, and other functional additives raises specific regulatory questions regarding potential migration into food, toxicological safety, and labeling requirements. The regulatory landscape for food contact materials varies significantly across different countries and regions, with the European Union, United States, and other major markets having distinct regulatory frameworks and approval processes. The lack of harmonized international standards for biopolymer packaging materials complicates global commercialization and increases the regulatory burden on manufacturers.

The safety evaluation of nanomaterials in food packaging has received particular attention due to concerns about the potential for nanoparticle migration and their biological effects. While many nanofillers, such as cellulose nanocrystals, are generally considered safe due to their derivation from food-grade materials, metal-based nanoparticles and metal–organic frameworks require careful toxicological assessment. The establishment of migration limits, analytical methods for detecting and quantifying nanoparticles in food simulants, and standardized toxicity testing protocols represent ongoing regulatory challenges. The biodegradability and compostability claims for biopolymer packaging materials require verification through standardized testing methods and certification by recognized organizations, adding to the regulatory complexity.

## 9. Future Perspectives and Research Directions

The field of biopolymer-based active and intelligent food packaging continues to evolve rapidly, with numerous opportunities for innovation and improvement. This section outlines promising future research directions and technological developments that could accelerate the transition from laboratory innovations to commercial reality.

### 9.1. Advanced Multi-Functional Integration

Future developments in biopolymer packaging will likely emphasize the integration of multiple functionalities within single materials or systems, moving beyond simple active or intelligent packaging toward sophisticated platforms that combine antimicrobial activity, antioxidant protection, quality monitoring, traceability, and consumer interaction. The development of packaging materials that can simultaneously inhibit microbial growth, prevent oxidative deterioration, monitor multiple quality parameters through different sensing mechanisms, communicate information to consumers through smartphone-readable indicators or RFID tags, and biodegrade safely at end of life represents an ambitious but achievable goal. The integration of electronic components and sensors with biopolymer substrates to create hybrid packaging systems that provide detailed information about storage conditions, temperature history, and quality status could enable more sophisticated supply chain management and consumer engagement.

The incorporation of multiple natural indicators responsive to different spoilage markers, such as pH-sensitive anthocyanins combined with ammonia-sensitive metal–organic frameworks and oxygen-sensitive dyes, could provide a more comprehensive and reliable quality assessment than single-indicator systems. The development of packaging materials with programmable or tunable release profiles for multiple active compounds, enabling sequential or triggered release in response to specific conditions, represents another frontier for advanced functionality. The creation of self-healing biopolymer packaging materials that can repair minor damage through reversible cross-linking or other mechanisms could enhance durability and extend useful life.

### 9.2. Artificial Intelligence and Machine Learning Applications

The application of artificial intelligence and machine learning approaches to biopolymer packaging development could significantly accelerate material discovery, optimization, and performance prediction. Machine learning models trained on databases of biopolymer properties, processing conditions, and performance outcomes could guide the design of new materials with desired characteristics, reducing the need for extensive trial-and-error experimentation. The use of computer vision and image analysis to interpret colorimetric indicator responses in intelligent packaging could enable automated quality assessment in supply chains and retail environments. The integration of packaging sensor data with machine learning algorithms for predictive modeling of shelf life and quality evolution could optimize inventory management and reduce waste [28].

The development of digital twins of packaged food products, incorporating models of food quality changes, packaging material properties, environmental conditions, and their interactions, could enable virtual testing and the optimization of packaging solutions before physical prototyping. The application of artificial intelligence to analyze consumer preferences, market trends, and sustainability metrics could guide the development of biopolymer packaging solutions that balance performance, cost, environmental impact, and consumer acceptance. The use of blockchain technology in combination with intelligent packaging for supply chain traceability and authentication represents another emerging area where digital technologies can enhance the value proposition of advanced packaging systems.

### 9.3. Sustainable Sourcing and Circular Economy Integration

Future developments must prioritize sustainable sourcing of biopolymer raw materials, ensuring that increased demand for packaging materials does not compete with food production, contribute to deforestation, or create other environmental or social problems. The utilization of agricultural waste streams, food processing by-products, and non-food biomass sources for biopolymer production represents a key strategy for sustainable sourcing. The development of integrated biorefineries that produce biopolymers alongside biofuels, biochemicals, and other value-added products from biomass feedstocks could improve economic viability while maximizing resource utilization. The cultivation of dedicated crops for biopolymer production on marginal lands unsuitable for food production, or the genetic engineering of crops to enhance biopolymer yield and properties, offers additional pathways for sustainable raw material supply.

The integration of biopolymer packaging into circular economy frameworks requires the development of appropriate end-of-life pathways including industrial composting, anaerobic digestion, or recycling systems specifically designed for biopolymers. The establishment of collection and processing infrastructure for biopolymer packaging, distinct from conventional plastic recycling streams, represents a critical need for realizing the environmental benefits of these materials. The design of biopolymer packaging materials with controlled biodegradation rates appropriate for their intended use duration and disposal pathway could optimize the balance between stability during use and rapid degradation after disposal [101,102,103,104]. The development of standardized testing methods and certification schemes for biodegradability and compostability in relevant environments, including soil, marine, and industrial composting conditions, would facilitate regulatory approval and consumer confidence.

### 9.4. Standardization and Regulatory Harmonization

The establishment of international standards for biopolymer packaging materials, covering aspects including performance requirements, testing methods, safety evaluation, biodegradability assessment, and labeling, would facilitate commercial development and global trade. The harmonization of regulatory requirements across different jurisdictions could reduce the burden on manufacturers and accelerate market entry for innovative packaging solutions. The development of rapid and cost-effective testing methods for evaluating key properties such as migration, antimicrobial efficacy, barrier performance, and biodegradation would support both regulatory compliance and quality control in manufacturing. The creation of databases and knowledge repositories containing information on approved materials, additives, and processing aids for biopolymer packaging would assist formulators and manufacturers in developing compliant products [105,106,107,108,109,110].

The establishment of clear guidelines for environmental claims and sustainability labeling of biopolymer packaging, preventing greenwashing while enabling the communication of genuine environmental benefits, represents an important need. The development of standardized life cycle assessment methodologies specifically tailored to biopolymer packaging, accounting for factors such as renewable resource use, biodegradability, and carbon sequestration, would enable more accurate comparison of environmental impacts compared to conventional materials. The creation of industry consortia or public–private partnerships to address common challenges, share best practices, and coordinate research efforts could accelerate progress toward the commercial viability of biopolymer packaging solutions.

### 9.5. Consumer Education and Market Development

The successful commercialization of biopolymer active and intelligent packaging requires consumer understanding, acceptance, and willingness to pay potential price premiums for enhanced functionality and environmental benefits. Consumer education initiatives explaining the benefits of biopolymer packaging, the proper use of intelligent packaging indicators, and appropriate disposal methods represent important components of market development. The demonstration of tangible benefits such as reduced food waste, improved safety, and environmental advantages through clear communication and labeling could drive consumer demand. The engagement of retailers, food manufacturers, and other supply chain stakeholders in promoting biopolymer packaging and creating demand pull represents a critical factor for market growth.

The development of business models that appropriately value the multiple benefits of advanced biopolymer packaging, including food waste reduction, environmental impact mitigation, and enhanced brand value, could justify higher costs and drive adoption. Targeting premium market segments, specialty products, or applications where conventional packaging performs poorly could provide initial market entry points for biopolymer solutions. The collaboration between packaging material developers, food manufacturers, retailers, and waste management companies to create integrated value chains for biopolymer packaging could address challenges across the entire lifecycle, from raw material sourcing through end-of-life disposal.

## 10. Conclusions

The development of biopolymer-based active and intelligent food packaging represents a convergence of materials science, food science, nanotechnology, and sustainability principles, addressing the urgent need for alternatives to conventional petroleum-based packaging while enhancing food preservation and quality monitoring capabilities. Recent research has demonstrated remarkable progress in creating sophisticated packaging systems from natural polysaccharides and proteins, enhanced through the incorporation of antimicrobial agents, colorimetric indicators, nanofillers, and advanced fabrication technologies. The starch-, chitosan-, cellulose-, pectin-, bacterial cellulose-, pullulan-, gelatin-, zein-, and dextran-based systems reviewed in this article illustrate the diverse palette of biopolymer materials available and the creative approaches being employed to overcome their inherent limitations while leveraging their unique properties.

The integration of multiple functionalities within single packaging materials, exemplified by the multifunctional starch–pectin–ZIF-67 microcrystalline cellulose films for pork preservation and the pH-responsive chitosan–chitin nanofiber films with anthocyanin indicators, represents a significant advancement over conventional passive packaging. These materials provide not only physical protection but also active preservation through antimicrobial and antioxidant activities, and real-time quality information through colorimetric indicators responsive to spoilage markers. The application of advanced processing technologies, including electrospinning, solution blow spinning, and cryogelation, enables the creation of nanostructured materials with enhanced surface area, controlled porosity, and sophisticated architectures such as core–shell nanofibers that facilitate controlled release and spatial organization of functional components.

Despite impressive progress, significant challenges remain regarding mechanical and barrier properties, the scalability of advanced fabrication techniques, cost competitiveness with conventional materials, stability during storage, and regulatory approval. The path toward commercial realization of biopolymer packaging solutions requires continued research addressing these challenges through innovative material design, process optimization, and comprehensive performance evaluation under realistic conditions. The development of packaging materials from agricultural waste streams such as coconut coir cellulose represents an important strategy for improving sustainability and economic viability while valorizing underutilized resources. The stabilization of sensitive functional compounds, such as anthocyanins through encapsulation in cyclodextrins and metal–organic frameworks, demonstrates the importance of protecting active components to ensure sustained functionality throughout the packaging lifecycle.

Future developments will likely emphasize the increasingly sophisticated integration of multiple functionalities, the incorporation of digital technologies and artificial intelligence for smart supply chain management, sustainable sourcing within circular economy frameworks, and standardization to facilitate regulatory approval and commercial adoption. The successful transition of biopolymer packaging from laboratory curiosity to mainstream commercial reality will require collaboration among researchers, industry, regulators, and consumers, along with continued innovation in materials, processing, and application development. The potential benefits of widespread adoption of biopolymer active and intelligent packaging are substantial, including reduced plastic waste accumulation, decreased food losses through improved preservation, enhanced food safety through real-time quality monitoring, and decreased environmental impact of the food packaging system. As research continues to address current limitations and demonstrate practical viability, biopolymer-based packaging solutions are poised to play an increasingly important role in creating a more sustainable and efficient food system for the future.

## Figures and Tables

**Table 1:** Key properties of different biopolymers in packaging materials.

Biopolymer Type	Material System	Modification (Substances and Amounts)	Improvements in Properties	Degradations or Limitations	Functionality and Target Product	Packaging Classification	Ref
Starch	Starch Cryogels and Hydrogels	Oxidation with sodium metaperiodate; loaded with diacetyl or mint essential oil	High water absorption capacity; controlled delivery of antimicrobial volatiles	Native starch requires chemical modification to achieve stability	Moisture control and microbial inhibition for fresh food	Active (Moisture Control and Antimicrobial)	[15]
Starch/Pectin	LSS/Pectin Composite Film	Loquat seed starch (LSS) + pectin (P) + 5% ZIF-67@MCC	Improved UV blocking, mechanical strength, and water/gas barrier; antibacterial activity	Complexity in synthesizing metal–organic framework fillers	Real-time monitoring and preservation of pork freshness	Active and Intelligent (Preservation and Freshness Indicator)	[26]
Starch	Cassava Starch/Fish Gelatin Film	5% cassava starch + 1:3 fish gelatin + 10% CEO nanoemulsion (CEON)	Extended fish filet shelf life to 10 days; high antioxidant and antibacterial properties	Increased film thickness and slight brown coloration	Preservation of fish filets	Active (Antimicrobial and Antioxidant)	[72]
Chitosan	Mesoporous Chitosan Nanofibers	Electrospinning with PEO (sacrificial); loaded with norfloxacin and phenylboronic acid	Rapid fluid adsorption (17 g/g); controlled antimicrobial release	High biodegradation rate (up to 30% mass loss) in specific pH	Bioabsorbable dressings for wound healing (transferable to food contact)	Active (Antimicrobial and Absorbent)	[38]
Chitosan	ChNF-reinforced Chitosan Films	Chitosan + chitin nanofibers (ChNFs) + 10 phr citric acid (CA) + anthocyanins	52.8% increase in gel content; enhanced thermal stability and pH color sensitivity	Partly soluble in acidic environments (47.2% mass loss for cross-linked films)	Freshness monitoring (pH-responsive) for various food items	Intelligent (Freshness Monitoring)	[43]
Cellulose	BC/PVA Composite Film	Bacterial cellulose (BC) + polyvinyl alcohol (PVA) + TiO_2_	Ultra-high oxygen barrier; UV resistance; oil and solvent resistance	Requires specific “brick sand” structure for optimal barrier	Sustainable preservation for general food items	Active (High-barrier and UV Protection)	[92]
Cellulose	CNC Biofilm from Coconut Coir	6:4 ratio of cellulose nanocrystals (CNC) to starch binder + cross-linker	High tensile strength (38.4 MPa) and elongation (8.2%)	Properties strictly dependent on the precise CNC/binder ratio	Sustainable alternative to plastic packaging	Active (Mechanical Support)	[98]
Cellulose	Taro Leaf-Inspired Coating	CNFs + alkyl ketene dimer (AKD) + CMPs + TiO_2_	Superhydrophobic and antibacterial; hierarchical surface roughness	Dependent on successful post-recrystallization of AKD	Food preservation and oil/water separation	Active (Water-repellent and Antimicrobial)	[51]

## Data Availability

No new data were created or analyzed in this study.
